# Structural Basis for Polyadenosine-RNA Binding by Nab2 Zn Fingers and Its Function in mRNA Nuclear Export

**DOI:** 10.1016/j.str.2012.03.011

**Published:** 2012-06-06

**Authors:** Christoph Brockmann, Sharon Soucek, Sonja I. Kuhlmann, Katherine Mills-Lujan, Seth M. Kelly, Ji-Chun Yang, Nahid Iglesias, Francoise Stutz, Anita H. Corbett, David Neuhaus, Murray Stewart

**Affiliations:** 1 Medical Research Council Laboratory of Molecular Biology, Cambridge CB2 0QH, UK; 2 Department of Biochemistry, Emory University School of Medicine, Atlanta, GA 30322, USA; 3 Department of Cell Biology, Sciences III, 1211 Geneva 4, Switzerland

## Abstract

Polyadenylation regulation and efficient nuclear export of mature mRNPs both require the polyadenosine-RNA-binding protein, Nab2, which contains seven CCCH Zn fingers. We describe here the solution structure of fingers 5-7, which are necessary and sufficient for high-affinity polyadenosine-RNA binding, and identify key residues involved. These Zn fingers form a single structural unit. Structural coherence is lost in the RNA-binding compromised Nab2-C437S mutant, which also suppresses the *rat8-2* allele of RNA helicase Dbp5. Structure-guided Nab2 variants indicate that *dbp5(rat8-2)* suppression is more closely linked to hyperadenylation and suppression of mutant alleles of the nuclear RNA export adaptor, Yra1, than to affinity for polyadenosine-RNA. These results indicate that, in addition to modulating polyA tail length, Nab2 has an unanticipated function associated with generating export-competent mRNPs, and that changes within fingers 5-7 lead to suboptimal assembly of mRNP export complexes that are more easily disassembled by Dbp5 upon reaching the cytoplasm.

## Highlights

► Nab2 Zn fingers 5-7 have an unusual coherent structure ► Nab2 has an unanticipated role in assembly of export-competent mRNPs ► NMR identifies Nab2 residues that bind polyadenosine ► Structure-guided mutants indicate Nab2's function in the gene expression pathway

## Introduction

Nuclear export of mRNA is the culmination of the nuclear phase of the gene expression pathway and appears to be mediated by a Brownian ratchet mechanism that involves three principal steps: formation of an export-competent mRNP (messenger ribonucleoprotein); translocation of this mRNP though nuclear pore complexes (NPCs); and disassembly of the export complex in the cytoplasm (Carmody and Wente, 2009; Grünwald et al., 2011; Stewart, 2007). Before export, nascent transcripts progress through a coordinated series of modifications, including 5′ capping, splicing, and 3′ cleavage/polyadenylation, that are mediated by a host of mRNA-binding proteins (Carmody and Wente, 2009; Grünwald et al., 2011; Kelly and Corbett, 2009; Iglesias and Stutz, 2008; Köhler and Hurt, 2007; Stewart, 2010). In budding yeast, nuclear export of bulk mRNA is mediated primarily by Mex67:Mtr2 that binds both mRNPs and NPC proteins (Carmody and Wente, 2009; Grünwald et al., 2011; Kelly and Corbett, 2009; Iglesias and Stutz, 2008; Köhler and Hurt, 2007; Stewart, 2010). Generation of export-competent mRNPs (reviewed by Carmody and Wente, 2009; Grünwald et al., 2011; Kelly and Corbett, 2009; Iglesias and Stutz, 2008; Köhler and Hurt, 2007; Stewart, 2010) is accomplished following termination of 3′ end processing and polyadenylation, in which the 3′ end-processing factor Pcf11 recruits the Yra1 adaptor that then recruits Mex67:Mtr2 (Johnson et al., 2009). This recruitment may be assisted by Nab2 (Batisse et al., 2009), a conserved polyadenosine-RNA-binding Zn finger (ZnF) protein required for both mRNA export and polyadenylation regulation (Anderson et al., 1993; Hector et al., 2002; Green et al., 2002; Marfatia et al., 2003; Kelly et al., 2007; Leung et al., 2009; Pak et al., 2011) that appears to become attached to the mRNP after splicing and during or immediately after polyadenylation (Batisse et al., 2009). After Mex67:Mtr2 binds, the mRNP is remodeled, during which Yra1 is removed (Lund and Guthrie, 2005), although Nab2 remains attached. Transport directionality is imposed by the RNA helicase, Dbp5, removing Mex67:Mtr2 in the cytoplasm, thereby preventing return of the mRNP to the nucleus (Alcázar-Román et al., 2006; Weirich et al., 2006; Cole and Scarcelli, 2006; Stewart, 2007). The *dbp5(rat8-2)* conditional allele (Snay-Hodge et al., 1998) has been used extensively to study mRNA nuclear export and is thought to encode Dbp5 with impaired helicase activity (Strahm et al., 1999; Suntharalingam et al., 2004; Tran et al., 2007; Zheng et al., 2010; Kelly et al., 2010).

How Nab2 contributes to mRNP assembly and disassembly is currently unclear. Nab2 appears to associate with the bulk of mRNAs before they are exported and although localized to the nucleus at steady state, it shuttles between the nucleus and cytoplasm (Green et al., 2002; Marfatia et al., 2003). The Nab2 protein contains four domains: an N-terminal PWI-like domain that interacts with NPCs (Suntharalingam et al., 2004; Zheng et al., 2010; Grant et al., 2008); followed by a Gln-rich linker; then an Arg-Gly (RGG) domain required for nuclear import (Aitchison et al., 1996); and finally a domain containing seven tandem CCCH ZnFs that binds polyadenosine-RNA in vitro and also contributes to polyA tail length control and the checkpoint for proper 3′ processing (Kelly et al., 2007, 2010). ZnFs 5-7 (ZnF5-7) are necessary and sufficient for high-affinity polyadenosine-RNA binding (Kelly et al., 2010). Nab2 remains attached to mRNPs during passage through NPCs and is removed at their cytoplasmic face by Dbp5 (Tran et al., 2007). Interestingly, mutation of the gene encoding the human Nab2 counterpart, *ZC3H14*, leads to an inherited form of intellectual disability (Kelly et al., 2007; Leung et al., 2009), highlighting the importance of this protein in the brain of higher organisms.

Here, we describe the solution structure of Nab2 ZnF5-7 and explore their interaction with polyadenosine-RNA. These three ZnFs have almost identical folds and, most unusually, associate with one another to form a single coherent structural unit. ZnF5-7 bind to eight consecutive adenines, and chemical shift perturbations (CSPs) identify residues on each finger that interact with RNA. These data, combined with the changes in affinity associated with mutation of key residues within each finger, indicate that the binding of ZnF6 to polyadenosine-RNA is different than that of fingers 5 and 7 and also indicate that Nab2 ZnFs function in the generation of export-competent mRNPs.

## Results and Discussion

### Nab2 ZnF5-7 Form a Single Domain

**Figure 1:**
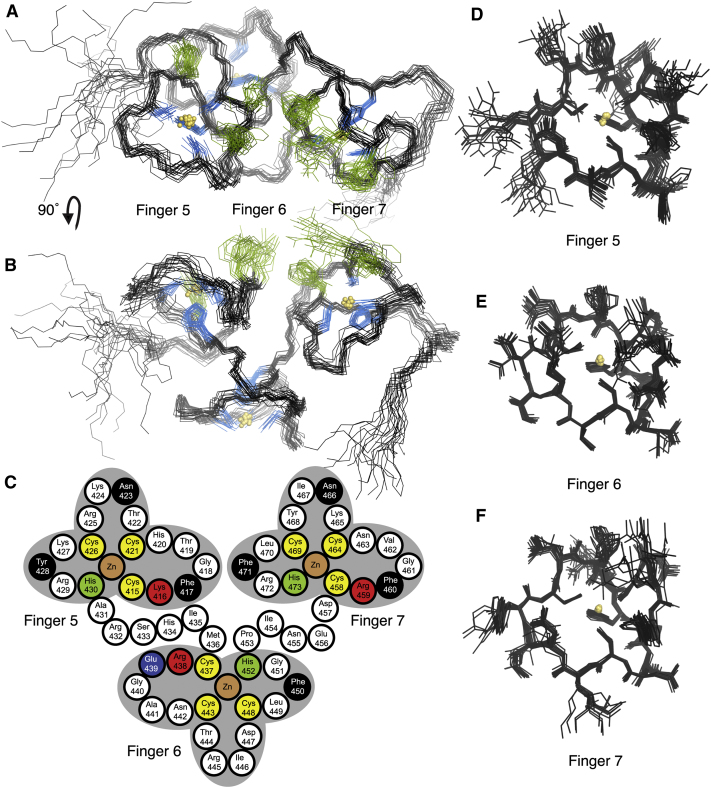
NMR Structure of Nab2 ZnF5-7 (A and B) Ensembles of the NMR structure of Nab2 residues 409–483. Of 50 calculated structures, 20 were selected by lowest total energy. The structures are superimposed based on the backbone of residues 410–480. Side chains of the Zn-coordinating residues are shown in blue. The RNA-binding residues on ZnFs 5 and 7 (see text) are shown in green. The three fingers associate to form a novel single structural domain in which individual fingers pack together to produce a pseudo-helical arrangement. (C) Schematic illustration of the structure of fingers 5-7. Three cysteines (yellow) and a histidine (green) bind the Zn ion (brown) in each finger. Isoleucines 435 and 454, located in the linkers between fingers, are buried in the interfaces between the fingers. Residues located in finger 5 (red, Lys 416; black, Phe417, Asn423, Tyr428) and finger 7 (red, Arg459; black, Phe460, Asn466, Phe471) show large chemical shift changes on addition of A_3_. Finger 6 has Glu439 (blue) instead of an aromatic residue in position 2. Mutation of Lys416 or Phe417 in finger 5, Cys437, Arg438, or Phe450 (black) in finger 6, or Arg459 or Phe460 in finger 7 decreases the affinity for polyadenosine (see Table 2). (D–F) The same ensemble of structures is shown as in (A) and (B) but is now shown superimposed on the Cα positions from the individual fingers: (D) shows ZnF5 containing residues 414–431, (E) illustrates ZnF6 containing residues 436–453, and (F) shows ZnF7 containing residues 457–474). See also Figure S1 and Movie S1.

The solution structure of a Nab2 fragment (residues 409–483) containing ZnF5-7 was determined using NMR spectroscopy (Figure 1; Table 1; see Movie S1 available online). A striking and unexpected feature of the structure was that the three ZnFs (residues 410–480) formed a single ordered domain (Figures 1A and 1B; Movie S1). The relative orientations of the ZnFs were defined by unambiguous interfinger NOE contacts. There were 69 contacts assigned between ZnFs 5 and 6 (mainly linking Lys410 to Ile446; Leu412 to Thr444 and Ile446; Gln414 and Lys416 to Ile454; and Ala431-Ser433 to Leu449-Gly451), 27 contacts between ZnFs 6 and 7 (mainly linking Met436 to Tyr468 and Leu470; and Ile454 to Leu470 and Phe471), and 14 contacts between ZnFs 5 and 7 (mainly linking Gln414, Gly418, and Ala431 to Leu470, and His434 to Tyr468). The spatial organization of ZnF5-7 resulted in the overall precision of the fragment when considered as a single entity (average backbone rmsd to the mean structure for residues 410–480 was 0.69 ± 0.15 Å) being comparable to those of the individual ZnFs. Average backbone rmsds to the mean structure for ZnF5 (residues 414–431), ZnF6 (residues 436–453), and ZnF7 (residues 457–474) were 0.45 ± 0.15 Å, 0.23 ± 0.04 Å, and 0.26 ± 0.08 Å, respectively (Figures 1A and 1B), and superpositions of the ensembles of individual ZnFs (Figures 1D–1F) were only marginally superior to the ensemble for the entire structure (Figures 1A and 1B). There may be some slight additional flexibility present within part of ZnF5, as evidenced by the higher rmsd for this finger and the slightly greater spread of structures in the Cys421-Cys426 loop.

Analysis of heteronuclear NOEs confirmed the relatively fixed relationship among the three ZnFs. Apart from a small number of residues at each end of the construct (N-terminal GPLGS-cloning artifact and residues 481–483), the backbone rigidity as evidenced by the heteronuclear NOE data was essentially uniform along the entire fragment, with no additional flexibility evident in the interfinger linker regions (Figure S1). ZnFs 5, 6, and 7 form an approximate right-handed helix, although the relative orientation between fingers 6 and 7 is not a precise recapitulation of that between fingers 5 and 6.

### Structure of Individual ZnFs

**Figure 2:**
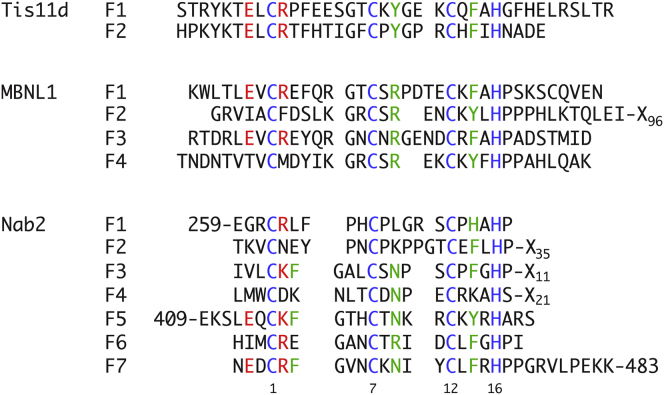
Alignment of the Sequences of Related ZnFs Shown are the seven ZnFs of Nab2 itself, as well as the closely related fingers from the proteins Tis11d and MBNL1. Residue positions within each finger are numbered taking the first cysteine residue as position 1. See also Figure S2.

**Figure 3:**
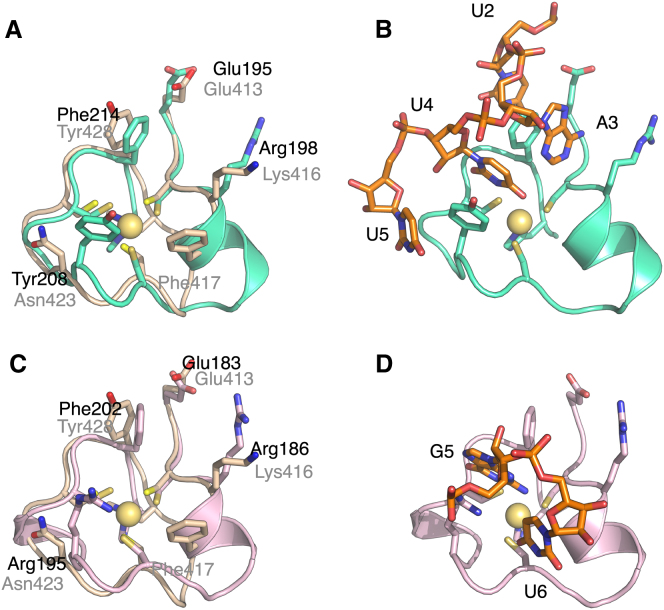
Comparison of Nab2 with Other Tandem ZnF Domains (A) Structural alignment of Nab2 ZnF5 (wheat) with ZnF2 of the Tis11d tandem ZnF domain (shown in cyan) (PDB 1RGO). Side-chain residues involved in RNA binding in the Tis11d structure are shown. (B) RNA (orange)-bound structure of Tis11d. (C) Structural alignment of Nab2 ZnF5 (shown as wheat) with ZnF3 of the MBNL1 tandem ZnF domain (shown in pink) (PDB 3D2S). Side-chain residues involved in RNA binding in chain (A) in the MBNL1 are shown. (D) RNA (orange)-bound structure of MBNL1.

Each finger has an identical CCCH pattern of Zn-ligating residues (Figure 2). For convenience we refer to residues in corresponding locations across the seven Nab2 ZnFs using a numbering scheme in which the first metal-binding Cys in each finger is assigned position 1 (Figure 2). Thus, the Zn-binding residues correspond to positions 1, 7, 12, and 16 (residues Cys415, Cys421, Cys426, and His430 in ZnF5; Cys437, Cys443, Cys448, and His452 in ZnF6; and Cys458, Cys464, Cys469, and His473 in ZnF7). In each finger the coordination by His is through N^ε2^ (Figure S2), and the absolute chirality of Zn-binding is R (see Berg, 1988). Compared to other ZnFs, those in Nab2 are very small, with each containing only 17 residues, and are devoid of secondary structure, consisting only of short loops running between the Zn-binding residues. All three fingers have similar structures (Figures 1 and 3; Movie S1), consistent with their sequence conservation (Figure 2). Their fold has some similarity to the ssRNA-binding tandem ZnF motifs found in TIS11d (Hudson et al., 2004) and MBNL1 (Teplova and Patel, 2008) (Figure 3A), although the short helix found between Zn ligands 1 and 2 in other tandem ZnF motifs is absent in Nab2, and there are generally two or three fewer residues between Zn ligands 1 and 2 in each of the Nab2 fingers. There are also structural differences in the region between Zn ligands 2 and 3, where the Nab2 ZnFs each contain four residues. The arrangement of the Zn-binding residues in the Nab2 ZnFs is in general very similar to that seen in TIS11d and MBLN1, although there is some difference in the χ2 angle for the histidine (approximately 90°–130° in Nab2 F5-F7 versus approximately 160°–180° in Tis11d and MBLN1). This difference might perhaps be related to the absence of other nearby aromatic side chains that might stack against the histidine (as occurs in the TIS11d and MBLN1 structures), or possibly to the different lengths of the interconnecting loops in Nab2; χ2 values of around 90° are not uncommon in other small ZnF proteins (e.g., PDB entries 2xox, 2kuo, 2kqb, 2kqc, and 3od8). The way in which Nab2 ZnF5-7 interact with one another to form a pseudo-helical arrangement (Figures 1A and 1B; Movie S1) is quite novel. In contrast the two fingers of TIS11d are structurally independent (Hudson et al., 2004), and in MBNL1 the head-to-tail arrangement of the two fingers is determined by a small antiparallel β sheet that forms between them (Teplova and Patel, 2008).

### RNA-Binding Surface of ZnF5-7

**Figure 4:**
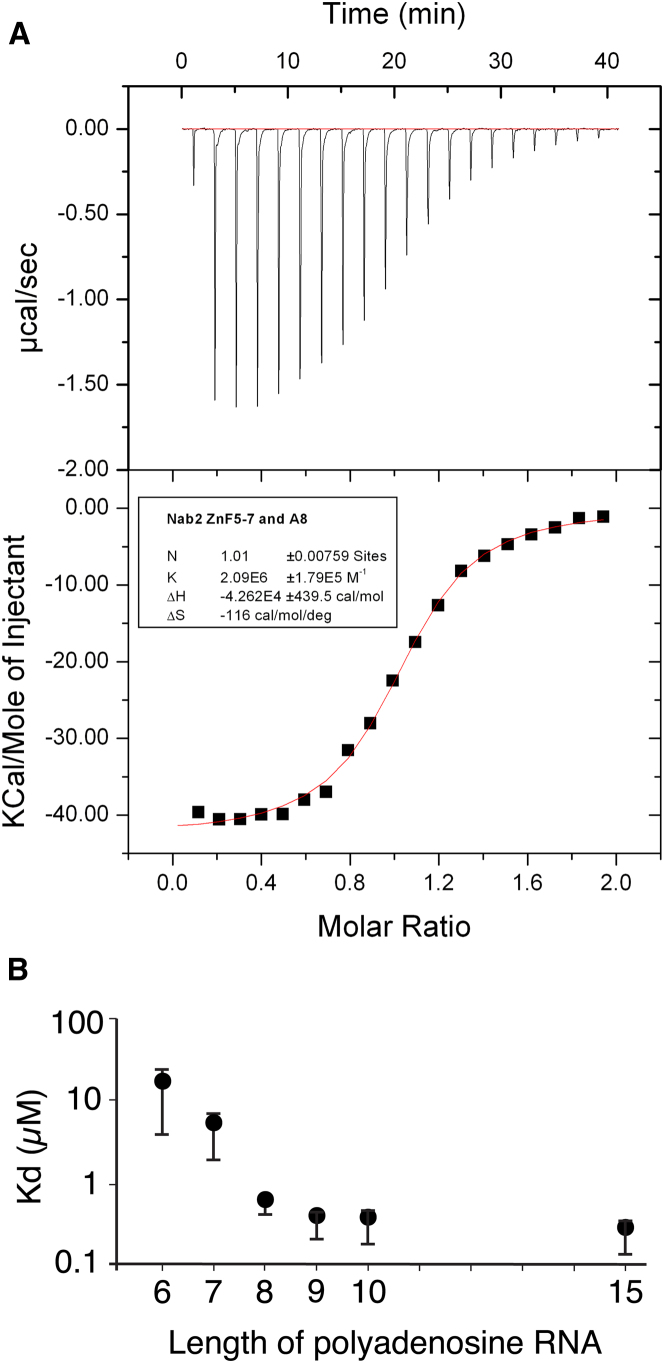
Affinity of Polyadenosine-RNA for Nab2 ZnF5-7 (A) ITC raw data (upper panel) for binding of A_8_ to Nab2 ZnF5-7 with integrated peaks and fitting curve (lower panel). (B) Influence of the length (A_6_–A_10_) of polyadenosine-RNA on the affinity for Nab2 ZnF5-7. Error bars represent SD from three independent measurements.

**Figure 5:**
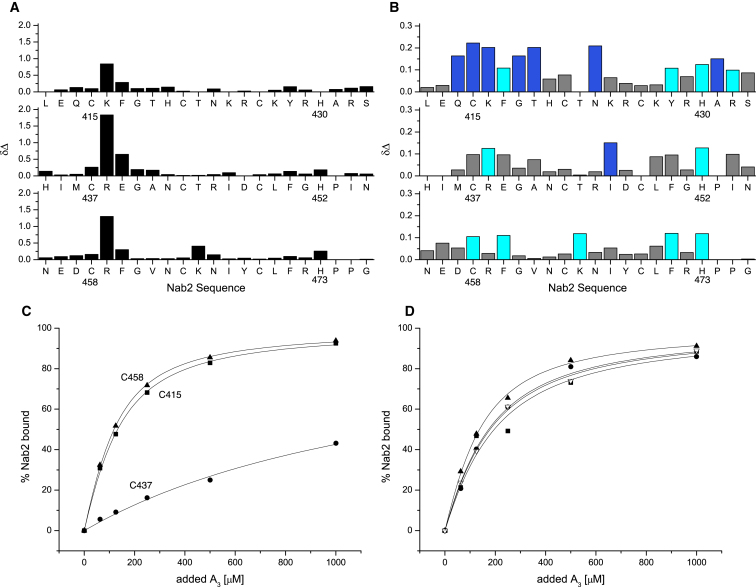
Binding of Nab2 ZnF5-7 to Polyadenosine-RNA (A) CSP of backbone amide groups of wild-type ZnF5-7 in the presence of 5 mM AMP. (B) CSP of backbone amide groups of wild-type Nab2 fingers 5-7 in the presence of 250 μM A_3_. (C) Normalized binding isotherms extracted from the titration data summarized in (B), following residues C415 (■), C437 (●), and C458 (▴). (D) Binding isotherms extracted from the titration of the E439F mutant with A_3_, following residues C415 (■), H420 (▿), C437 (●), and C458 (▴).

Determination of the affinity of progressively longer polyadenosine-RNAs using isothermal calorimetry (ITC) indicated that Nab2 ZnF5-7 bound eight adenines (Figure 4). Thus, the affinity of A_6_ and A_7_ was 14 μM but increased in strength to the order of 100 nM for A_8_, A_9_, and A_10_. Although chemical shift titration experiments with RNA containing more than three adenines were difficult to interpret because of line broadening, for AMP and A_3_ the extent of line broadening caused by complex formation with Nab2 ZnF5-7 was sufficiently low to enable high-resolution measurements to be made, possibly because the lower affinities of these ligands moved the exchange rates into a faster regime that was more favorable for detection of NMR signals. Figures 5A and 5B show amide CSP experiments in which either AMP or A_3_ was added to ZnF5-7. With AMP, all three fingers showed the strongest perturbation at a highly conserved basic residue adjacent to the first Zn-binding Cys (position 2; Lys416, Arg438, Arg459) and its immediate neighbors (Figures 1C, 2, and 5A). The response to A_3_ binding was somewhat less clear-cut and involved additional residues (Figure 5B), consistent with the larger ligand forming a larger and more complex set of interactions. The largest and most extensive perturbations were seen for finger 5, and as seen with AMP, there were large perturbations at or near the basic residue at position 2 for all three fingers, albeit now affecting a larger group of residues in this region. There were additional large perturbations at sites at or near position 9 (Asn423, Ile446, Lys465) and the conserved aromatic residue at position 14 (Tyr428, Phe450, Phe471). Generally, these residues are conserved among the seven Nab2 ZnFs, especially the basic residue at position 2 and the aromatic at position 14 (Figure 2). For some other positions conservation is more limited and is restricted to ZnFs 3, 5, and 7. This trend is particularly clear for the aromatic residue at position 3, which becomes a charged residue in ZnFs 2, 4, and 6, and to a lesser extent for the Asp at position 9.

Approximate binding constants for the individual fingers of ZnF5-7 derived from the A_3_ CSP data (Figure 5C) indicated that ZnF6 has a markedly lower binding affinity for A_3_ than does ZnF5 or ZnF7. Sequence comparisons indicated that the lower affinity exhibited by ZnF6 might be due to the absence of an aromatic residue at position 3 (which is instead Glu at position 439). Consistent with this hypothesis, the Nab2-E439F variant restored the affinity of ZnF6 to levels comparable to those of fingers 5 and 7 (Figure 5D). Overall, these results indicate that, although fingers 5 and 7 interact with polyadenosine-RNA in similar ways, finger 6 appears to contribute to binding in a different way. The 7 Nab2 ZnFs probably bind 20–25 adenosines in vitro (Viphakone et al., 2008). Although the 3 high-affinity binding ZnFs of Nab2, ZnF5-7, bind to a stretch of 8 adenosines, fingers 1–4 exist as 2 closely spaced pairs separated by a 39-residue linker and so are unlikely to form a compact structure similar to that observed for fingers 5-7. Thus, an estimate of an additional 12–17 nucleotides bound by fingers 1–4 in addition to the 8 nucleotides bound by fingers 5-7 seems quite consistent with the measurements obtained for all 7 fingers.

### Nab2-C437S Destabilizes ZnF6, and Structural Coherence between ZnF5 and ZnF7 Is Lost

**Figure 6:**
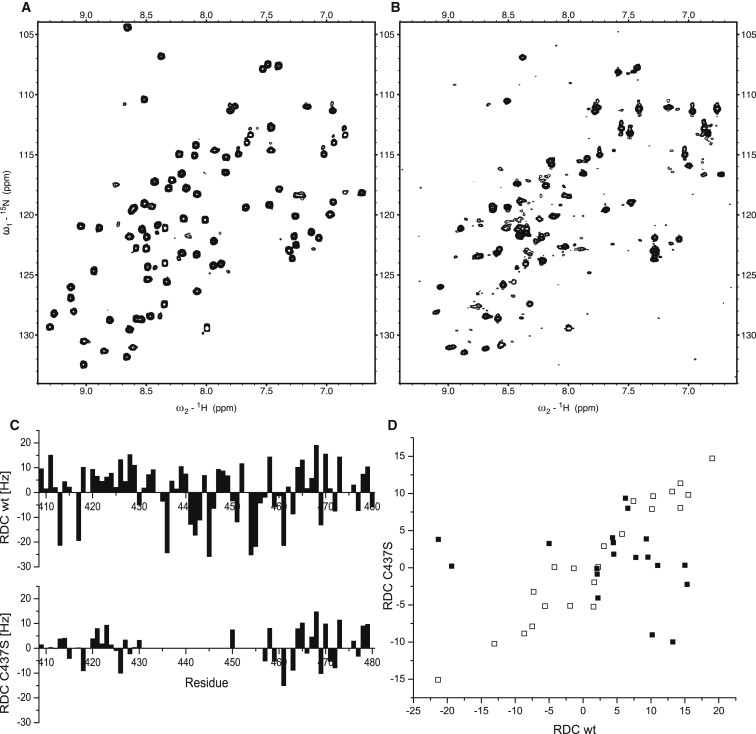
Effects of the C437S Mutant on the Structure of Nab2 ZnF5-7 (A) ^15^N-HSQC spectrum of wild-type Nab2^409–483^. (B) The same spectrum as shown in (A) but for the C437S Nab2 mutant. Comparison of (A) and (B) showed that peaks close to the original positions of the finger 5 and 7 amides are retained, consistent with these fingers still being folded, whereas most of the peaks from ZnF6 are significantly broadened, probably indicating exchange between different conformations. (C) Comparison of backbone ^1^H-^15^N RDC values obtained for the wild-type and C437S Nab2 proteins. The absence of measurable values for ZnF6 reflects the broad or unresolved nature of the corresponding peaks in these cases, whereas the reduced magnitudes of the RDCs in ZnF7 and (especially) ZnF5 indicate a higher degree of relative mobility for these fingers in the mutant. (D) Correlation plot of N-H RDC values obtained for the wild-type and C437S Nab2 proteins. RDC data obtained for ZnF5 (residues 410–430) are shown using filled squares (■), whereas RDC data obtained for ZnF7 (residues 458–479) are shown using open squares (□). A strong correlation is visible between the RDCs measured for the wild-type and the C437S mutant for ZnF7, but such a correlation is absent for the corresponding RDC data for ZnF5. This suggests that the alignment is dominated by effects involving ZnF7 in both the wild-type and mutant proteins but that in the mutant, ZnF5 is mobile relative to ZnF7, thereby rendering the alignment for ZnF5 in the mutant independent of that for ZnF7.

The importance of the structural coherence among ZnF5-7 is underlined by Nab2 variants in which Cys437, one of the Zn-binding residues of ZnF6, is mutated (to Ala, Ser, or Arg). These variants bind polyadenosine-RNA more weakly than does wild-type Nab2, and they suppress the *dbp5(rat8-2)* phenotype (Tran et al., 2007; Kelly et al., 2007, 2010). Mutation of Cys437 would be expected to destabilize ZnF6 because a Zn-binding residue is altered. Direct comparison of the ^15^N-HSQC spectra of wild-type and C437S-Nab2 ZnF5-7 (Figures 6A and 6B) showed that peaks close to the original positions of the finger 5 and 7 amides are retained, consistent with these fingers still being folded, whereas most of the peaks from ZnF6 are significantly broadened, indicating exchange between different conformations. The comparison of ^1^H-^15^N RDC data from the wild-type construct and from the mutant indicates that Nab2-C437S has a higher degree of relative mobility because RDCs of fingers 5 and 7 are significantly reduced in magnitude for the mutant (Figure 6C). In addition a strong correlation was seen between RDCs measured for ZnF7 in the wild-type and those measured in the C437S mutant, whereas a similar correlation was not seen for RDCs measured for ZnF5 (Figure 6D). This finding suggests that the alignment to the medium may be dominated by ZnF7 in both cases, but because the orientation of ZnF5 is no longer rigidly maintained relative to that of ZnF7 in the C437S mutant, the RDCs observed at ZnF5 in the mutant become markedly different than those in the wild-type protein. Taken together, these results indicate that in C437S-Nab2, fingers 5 and 7 retain their structure, whereas ZnF6 is destabilized, leading to an enhanced flexibility of ZnF5 relative to ZnF7. Significantly, the affinity of the C437S variant for A_8_ was reduced ∼40-fold to 19 μM (Table 2), consistent with the structural coherence of ZnF5-7 being important for the binding to polyadenosine-RNA.

### Engineered Nab2 Variants Confirm the CSP Results

The role of the interactions detected using CSPs was explored using a range of engineered Nab2 variants. ZnF6 differs from fingers 5 and 7 in having Glu rather than Phe in position 3 (Figure 2), and NMR titrations indicated that ZnF6 had substantially reduced affinity for A_3_ relative to fingers 5 or 7 (Figure 5C). The importance of position 3 was confirmed by interchanging these residues. Mutating Phe in position 3 to Glu in either finger 5 or 7 (F417E and F460E, respectively) reduced the affinity of ZnF5-7 for A_8_ by roughly 40-fold, and simultaneous mutation of both Phe417 and Phe460 reduced affinity further (Table 2). Mutation of the Glu of ZnF6 to Phe (E439F) increased affinity for A_8_ marginally (Table 2) (although for binding of A_3_ the corresponding increase was greater; Figure 5B). Decreases in affinity for A_8_ to ∼10–50 μM were observed following mutation of the basic residues in position 1 of each finger (K416A, R438A, or R459A), albeit the decrease in affinity seemed to be about 2-fold greater for ZnF6, consistent with its making a different contribution to polyadenosine-RNA binding than fingers 5 and 7. The affinity of C437S was similar to that observed for F417E or F460E and was consistent with each finger in this variant now binding polyadenosine-RNA independently rather than in concert as in the wild-type.

### Functional Impact of Mutations in Nab2 Fingers 5-7

**Figure 7:**
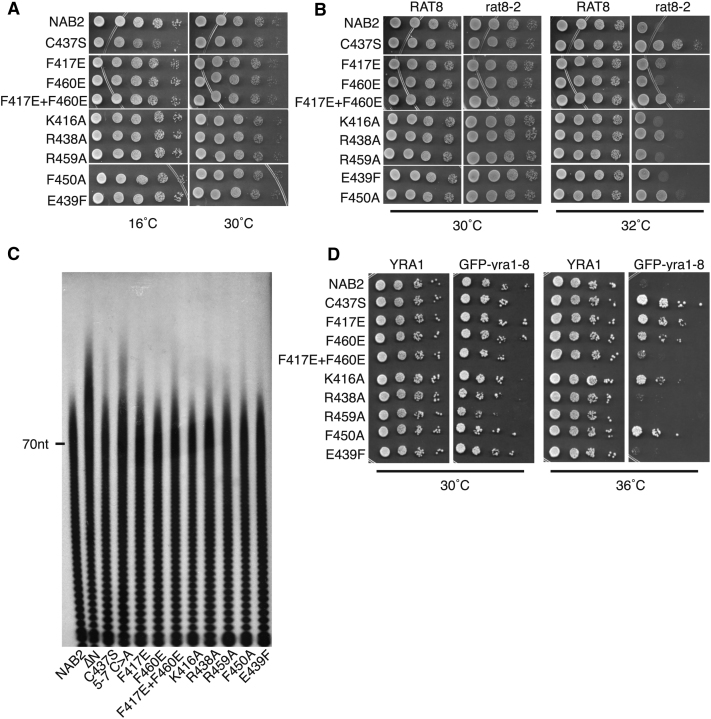
Functional Analysis of Nab2 Variants In Vivo The function of Nab2 variants was assessed using a plasmid-shuffle assays such that each variant was examined as the only functional copy of the essential Nab2 protein. Results of these experiments are summarized in Table 2. For growth assays, yeast cells expressing each Nab2 variant were serially diluted and spotted on plates. (A) Nab2 ZnF variants can function in place of Nab2. Each variant supports normal cell growth at 30°C, but *nab2-C437S* shows slow growth at 16°C, indicating a cold-sensitive growth phenotype. (B) Suppression of the temperature-sensitive growth phenotype of *dbp5(rat8-2)* mutant cells by Nab2 variants. A plasmid-shuffle assay in Δ*NAB2 rat8-2* mutant cells was employed to examine suppression of the temperature-sensitive growth of *dbp5(rat8-2)* mutant cells at 32°C. As controls, no suppression is observed with wild-type *NAB2*, whereas *nab2-C437S* suppresses robustly. (C) Bulk poly(A) tail length was examined by an RNaseA/T1 assay. Cells expressing each Nab2 variant as the sole copy of Nab2 were grown to log phase, and poly(A) tails were labeled and resolved by gel electrophoresis. The position of a 70-nucleotide (70nt) marker is indicated. (D) Suppression of the temperature-sensitive growth phenotype of GFP-*yra1-8* mutant cells. A plasmid-shuffle assay in Δ*NAB2* GFP-*yra1-8* mutant cells was employed to examine suppression. As a control, no suppression is observed with wild-type *NAB2*. In contrast, *nab2-C437S* robustly suppresses, and some but not all of the variants suppress the temperature-sensitive growth of GFP-*yra1-8* cells at 36°C. See also Figure S3.

The role of specific residues within Nab2 ZnF5-7 was examined by engineering amino acid substitutions in Nab2 and assessing their function in yeast lacking the endogenous essential *NAB2* gene using a plasmid-shuffle assay (Table 2). None of the individual residues examined was absolutely required for the essential function of Nab2 because in each case cell growth was identical to wild-type cells at 30°C (Table 2; Figure 7A). Previous work showed that the C437S mutation, which decreases the affinity of Nab2 for polyadenosine-RNA, suppresses the temperature-sensitive phenotype of the *rat8-2* allele of *DBP5*, which remodels the mRNP at the NPC cytoplasmic face (Tran et al., 2007; Kelly et al., 2010). Significantly, several engineered Nab2 ZnF variants showed at least some suppression of the temperature-sensitive growth of *dbp5(rat8-2)* (Table 2; Figure 7B). Although C437S-Nab2 was the strongest suppressor, variants in ZnF6 (C437S, R438A, F450A) that had reduced affinity for A_8_ also showed some suppression. However, several ZnF5 and ZnF7 variants that had similarly reduced affinity (K416A, R459A, F460E) did not show suppression of *dbp5(rat8-2)*. The only variant not located in ZnF6 to show substantial suppression combined changes in both fingers 5 and 7 (F417E+F460E double mutant) and had very low affinity for polyadenosine-RNA. Overall, these findings are not consistent with the hypothesis (Tran et al., 2007) that simply decreasing the affinity of Nab2 for polyadenosine-RNA leads to suppression of the temperature-sensitive growth of *dbp5(rat8-2)* cells. Although a minimum threshold affinity for polyadenosine-RNA (stronger than ∼20 μM) might be necessary for suppression of *dbp5(rat8-2)*, the most consistent correlation was with mutations located in ZnF6.

Several Nab2 ZnF variants generated mRNAs with longer polyA tails (Figure 7C; Table 2). However, although some variants (K416A, R459A, F460E) in which the affinity for polyadenosine-RNA in vitro was reduced to ∼20 μM retained wild-type polyA tail length control, there was a stronger correlation between suppression of *dbp5(rat8-2)* and hyperpolyadenylation, both of which were observed for the C437S, R438A, F450A, and F417E+F460E variants (Table 2). As observed previously for Nab2-C437S and -F450A (Kelly et al., 2010), none of the Nab2 ZnF variants tested showed aberrant nuclear accumulation of polyA-RNA, indicating that they are not rate limiting for polyA-RNA export (Figure S3).

In addition to suppressing *dbp5*(*rat8-2)*, many of the engineered Nab2 ZnF variants also suppressed the *GFP*-*yra1-8* mutant (Vinciguerra et al., 2005) (Table 2; Figure 7D). Yra1p functions as an adaptor for recruiting Mex67:Mtr2, but it is removed from mRNP complexes prior to exit from the nucleus (Lund and Guthrie, 2005). Again, *nab2-C437S* showed the strongest suppression, and generally, variants that showed some suppression of *dbp5(rat8-2)* also suppressed *GFP*-*yra1-8* and showed altered polyA tail length. Strikingly, these defects do not correlate simply with a decreased affinity for polyadenosine-RNA, indicating that Nab2 has an additional function early in the mRNA export pathway associated with the generation of export-competent mRNPs.

### Implications for mRNA Nuclear Export

The in vivo results obtained using structure-based variants indicate that, in addition to controlling polyA tail length, Nab2 functions in the generation of export-competent mRNPs. Nuclear mRNA export is terminated by the removal of Mex67:Mtr2 by Dbp5 at the NPC cytoplasmic face (Lund and Guthrie, 2005; Alcázar-Román et al., 2006; Weirich et al., 2006; Cole and Scarcelli, 2006; Stewart, 2007). Because *nab2-C437S* suppresses the temperature-sensitive growth of *dbp5(rat8-2)* cells, it has been suggested that Dbp5 could remove Nab2 as well as Mex67:Mtr2 from the mRNP (Tran et al., 2007). This hypothesis was supported by the finding that Nab2-C437S binds more weakly to polyadenosine-RNA than does wild-type and is more readily removed from RNA by Dbp5 (Kelly et al., 2007; Tran et al., 2007). However, this weaker binding of Nab2-C437S to polyadenosine-RNA does not appear to explain its suppression of *dbp5(rat8-2)* because, as shown in Table 2, we generated several other Nab2 variants that bind polyadenosine-RNA with affinity comparable to that of Nab2-C437S, but which do not suppress the *dbp5(rat8-2)* allele. Therefore, suppression of *dbp5(rat8-2)* by *nab2* alleles does not seem to stem directly from removal of Nab2 at the NPC cytoplasmic face. Instead, the suppression of *dbp5(rat8-2)* by *nab2* alleles may derive from suboptimal mRNP assembly within the nucleus generating a configuration that can be more easily disassembled by Dbp5 to remove Mex67:Mtr2. In principle, Nab2-C437S could influence mRNP export complex disassembly either by increasing the efficiency of disassembly, or by weakening the binding of Mex67:Mtr2 to the mRNP, either through an impaired adaptor function or by generating an aberrant export complex that is less stable than wild-type. Although the activity of Dbp5 can be augmented by Gfd1 (Snay-Hodge et al., 1998), which appears to function as a scaffold to increase the local concentration of the components of the mRNP disassembly machinery (Suntharalingam et al., 2004; Zheng et al., 2010), it is not easy to see how mutating the Nab2 ZnFs could enable Gfd1 to function in this way. Similarly, although Nab2 may also act as an adaptor to recruit Mex67:Mtr2 to the mRNP (Iglesias et al., 2010), the interaction between these proteins involves the Nab2 RGG domain, and not the ZnFs. Taken together, these results indicate that it is unlikely that the suppression of *dbp5(rat8-2)* by the ZnF6 C437S mutation results from a direct effect in the cytoplasm.

Although the affinity of the Nab2 variants for RNA did not correlate with genetic suppression of *dbp5(rat8-2)*, this suppression correlated more closely with impaired control of polyA tail length and also with suppression of the GFP-*yra1-8* allele (Table 2; Figures 7C and 7D). Because Nab2 is thought to modulate polyA tail length within the nucleus, this correlation suggests that the suppression of *dbp5(rat8-2)* may be closely linked to events that occur in the nucleus and render the resulting mRNP easier to disassemble in the cytoplasm, rather than directly facilitating the removal of Nab2 by Dbp5. Consistent with Nab2 influencing the generation of export-competent mRNPs in the nucleus as well as polyadenylation, the addition of Nab2 and Yra1 to the mRNP occurs at the termination of polyadenylation and is influenced by components of the cleavage and polyadenylation factor (CPF) such as Pcf11 (Johnson et al., 2009, 2011). In this context it is significant that *nab2* mutants exacerbate the growth defect of the GFP-*yra1-8* allele, whereas overexpression of *NAB2* suppresses this phenotype (Iglesias et al., 2010; Vinciguerra et al., 2005), and moreover, Yra1 stimulates the interaction between Nab2 and Mex67 (Iglesias et al., 2010). Because Yra1 is removed from mRNPs before export (Lund and Guthrie, 2005), these observations strongly suggest that Nab2 participates together with Yra1 in the generation of export-competent mRNPs. Although the specific steps leading to generation of export-competent mRNPs have not all been defined, there appears to be a remodeling of the mRNP that is coupled to the termination of polyadenylation (Johnson et al., 2009, 2011; Qu et al., 2009; Rougemaille et al., 2008) by which stage Nab2 has been added (Batisse et al., 2009). This remodeling results in the removal of Yra1 from the mRNP coupled with attachment of Mex67:Mtr2. Although some of our *nab2* variants also generate longer polyA tails, it is unlikely that this is related to the suppression of *dbp5(rat8-2)* because mutations in *SAC3* and *APQ12* that generate longer tails (Baker et al., 2004) are synthetically lethal with *dbp5(rat8-2)* (Scarcelli et al., 2008). Because Yra1 functions in the generation of export-competent mRNPs and is removed from them prior to export, our finding that a range of *nab2* variants, including *nab2-C437S*, suppresses the conditional growth of *GFP-yra1-8* cells (Table 2; Figure 7D) strongly supports the hypothesis that Nab2 plays a key role in the nucleus in ensuring proper assembly of an export-competent complex. Therefore, this result would be more consistent with the *nab2-C437S*-mediated suppression of the *dbp5(rat8-2)* allele deriving from production of suboptimally assembled mRNPs in which Mex67:Mtr2 can be more easily removed by Dbp5-mediated remodeling at the NPC cytoplasmic face rather than weakening the direct interaction between Nab2 and Mex67 or RNA.

In summary, Nab2 ZnF5-7 have a novel conformation in which they form a single coherent structural unit that binds eight consecutive adenosines. Both chemical shift measurements and engineered mutations indicate that all three fingers bind to polyadenosine-RNA, albeit the way in which ZnF6 binds appears to be different than that seen for fingers 5 and 7. Engineered mutations guided by the structure of fingers 5-7 indicated that basic residues in position 1 were important for the interaction, as were aromatic residues in position 2 of fingers 5 and 7, together with Phe450 in ZnF6. The way in which these mutations influenced polyA tail length and cell growth in *dbp5(rat8-2)* or *GFP*-*yra1-8* backgrounds indicates that Nab2 functions in the generation of export-competent mRNPs and that specific changes within fingers 5-7 generate mRNPs that, once they reach the cytoplasm, are disassembled more readily by Dbp5 than wild-type mRNPs.

## Experimental Procedures

### Cloning and Protein Purification

*S. cerevisiae* Nab2 residues 409–483 were cloned into the BamHI and XhoI sites of pGEX6P-1 (GE Healthcare), which resulted in an additional N-terminal sequence GPLGS being retained on the final protein. The plasmid was transformed into *E. coli* strain BL21 DE3, and cells were grown in M9 minimal medium at 37°C to an optical density (OD) of 0.6. Protein expression was induced by addition of 200 μM IPTG and 250 μM ZnCl_2_. Expression was carried out at 20°C overnight. The protein was then purified using standard GST-purification methods in 50 mM Tris (pH 8.0), 200 mM NaCl, 10 μM ZnCl_2_, and 5 mM β-mercaptoethanol. The GST-fusion protein was eluted using reduced glutathione and cleaved overnight at 4°C using “PreScission” 3C-protease (GE Healthcare). The cleaved protein was concentrated and subjected to gel filtration chromatography on a S75 column (GE Healthcare) in 50 mM Tris-HCl (pH 6.75), 50 mM NaCl, 50 mM Glu/Arg, 10 μM ZnCl_2_, and 5 mM β-mercaptoethanol. Fractions containing the desired Nab2 fragment were pooled and concentrated up to 1.2 mM. Nab2 variants were generated using site-directed mutagenesis and cloned into the same vector. The mutants were purified using the same protocols as for the native protein.

### NMR Spectroscopy and Structure Calculations

Data were acquired at 17°C on Bruker DMX600 and DRX500 spectrometers, each equipped with a triple-resonance (^1^H/^15^N/^13^C) cryoprobe. NMR samples contained 50 mM Tris-HCl (pH 6.75), 50 mM NaCl, 50 mM Glu/Arg, 10 μM ZnCl_2_, 5 mM β-mercaptoethanol, and 5% D_2_O. Inclusion of the Arg/Glu mixture was necessary to obtain acceptable line widths, presumably because it reduced aggregation (Hautbergue and Golovanov, 2008). Although detection of NOE cross peaks was inhibited in the immediate region of the Arg/Glu ^1^H signals, this did not interfere with establishing the structure (Table 1). ^1^H, ^15^N, and ^13^C chemical shifts were calibrated using sodium 3,3,3-trimethylsilylpropionate (TSP) as an external ^1^H reference (Wishart et al., 1995).

Unless stated otherwise, all NMR experiments for the free protein were performed using ^15^N- or ^15^N, ^13^C-labeled protein samples. As described in Supplemental Experimental Procedures, an essentially complete set of resonance assignments was made using a standard suite of triple-resonance NMR experiments, and structural constraints were derived from NOESY and RDC data. Structures were calculated using a three-stage protocol. First, the semiautomatic program CYANA (Herrmann et al., 2002) was used to make initial NOE cross-peak assignments, then the program XPLOR-NIH (Schwieters et al., 2003) was used to iteratively refine the NOE constraint list and to allow specific Zn-binding terms to be employed in the force field, and lastly the program AMBER 9 (Case et al., 2006) was used for final refinement with a full force field and an implicit water-solvent model (Xia et al., 2002). Details are given in Supplemental Experimental Procedures. The program CLUSTERPOSE was used to calculate the mean rmsd of ensembles to their mean structure (Diamond, 1995).

### NMR Chemical Shift Titrations

[^1^H, ^15^N]HSQC spectra were acquired at 24°C or 17°C using Bruker DMX600 and DRX500 spectrometers during stepwise addition of either AMP or A_3_ to ^15^N-labeled wild-type Nab2 fingers 5-7 or the E439F variant in 50 mM Tris-HCl (pH 6.75), 50 mM NaCl, 50 mM Glu/Arg, 10 μM ZnCl_2_, and 5 mM β-mercaptoethanol at a concentration of 100 μM for A_3_ and 200 μM for AMP. Spectra were processed using XWINNMR (Bruker BioSpin GmbH, Karlsruhe, Germany) and visualized in Sparky (Goddard and Kneller, 2008). ^15^N-HSQC spectra were recorded with the Nab2 sample alone and then after stepwise addition of AMP to 6.25, 12.5, 25, 50 mM, or A_3_ to 62.5, 125, 250, 500, 1,000 μM. The CSP data shown in Figure 5 for the Nab2 fragments binding to A_3_ and AMP were obtained for each residue by comparing the amide chemical shift values of ^15^N-Nab2 fingers 5-7 alone with those of ^15^N-Nab2 fingers 5-7 in the presence of a 2.5-fold molar excess A_3_ and a 250-fold excess of AMP. Values were calculated using the equation

$$\text{CSP}=\sqrt{\left({\left(0.2\Delta \text{δN}\right)}^{2}+\left(\Delta {\text{δH}}^{2}\right)\right)},$$

and apparent binding constants for the individual fingers were extracted from the A_3_ data by following residues Cys415, Cys437, Cys458, and His420 using a model for one-site binding, neglecting ligand depletion from other binding sites (Wintjens et al., 2001).

### ITC

ITC measurements were performed using a MicroCal iTC200 with ZnF5-7 and polyadenosine-RNA of different defined lengths, dialyzed against 50 mM Tris-HCl (pH 8.5), 50 mM NaCl, 10 μM ZnCl_2_, and 5 mM β-mercaptoethanol. In a typical experiment, 30 μM polyadenosine-RNA was pipetted into the sample cell containing a 200 μM protein solution. Pipetting 30 μM RNA into buffer showed a negligible change, and so corrections were unnecessary. Protein and RNA concentrations were determined by extinction at 280 and 260 nm, respectively.

#### Chemicals, Plasmids, and S. cerevisiae Manipulations

Chemicals were obtained from Fisher Scientific (Pittsburgh), Sigma-Aldrich (St. Louis), or US Biological (Swampscott, MA, USA) unless otherwise noted. DNA manipulations were performed according to standard methods (Sambrook et al., 1989), and all media were prepared by standard procedures (Adams et al., 1997). *S. cerevisiae* strains and plasmids are described in Table S1. Plasmids encoding Nab2 variants were generated by site-directed mutagenesis of a wild-type *NAB2* plasmid (pAC717) using the QuikChange Site-Directed Mutagenesis Kit (Stratagene). All plasmids were fully sequenced.

#### In Vivo Functional Analysis

The in vivo function of each Nab2 mutant was tested using a plasmid-shuffle assay (Boeke et al., 1987). *S. cerevisiae* cells deleted for *NAB2* (ACY427) and containing a wild-type *NAB2 URA3* plasmid (pAC636) were transformed with *LEU2* plasmids expressing various Nab2 mutants. Transformants were grown to saturation and then cells were plated on 5-Fluoroorotic acid (5-FOA) to select for loss of the wild-type maintenance plasmid. These cells were then grown to saturation, normalized for cell number, and serially diluted and spotted onto ura^−^ leu^−^ glucose plates. Plates were then incubated at 18°C, 25°C, 30°C, or 37°C for 3–5 days. For analysis of either *dbp5*(*rat8-2)* or *GFP-yra1-8* suppression, a plasmid-shuffle assay was performed as described by Tran et al. (2007). Briefly, *ΔNAB2 rat8-2* or *ΔNAB2 GFP-yra1-8* cells transformed with plasmids expressing wild-type or mutant Nab2 were first grown on selective media containing 5-FOA, then grown on rich media (YPD), and finally grown to saturation, serially diluted, and spotted onto YPD. Plates were then incubated at 16°C, 25°C, 30°C, 32°C, or 36°C for 3–5 days.

#### PolyA Tail Length

Cells expressing wild-type or mutant Nab2 proteins were inoculated into YPD media and grown to saturation at 30°C. Cells were then diluted into 50 ml of YPD and grown at either 30°C or 16°C until they reached OD_600_ of 0.4–0.6. Twenty OD units of cells was harvested from each culture, and polyA tail length was determined as described by Minvielle-Sebastia et al. (1991) and Chekanova and Belostotsky (2003). Briefly, total RNA was end labeled with ^32^P-pCp and T4 RNA ligase, after which it was digested with RNases A/T1 to remove non-polyA-RNA, and the ^32^P-labeled RNA was then ethanol precipitated. Resuspended RNA was then resolved by denaturing urea-acrylamide gel electrophoresis and imaged using a phosphoimager.

#### Fluorescence In Situ Hybridization

Cells expressing wild-type Nab2 or Nab2 mutant proteins were initially grown in 2 ml cultures to saturation at 30°C. These starter cultures were then used to inoculate 10 ml cultures that were grown overnight (approximately 12–16 hr) at 30°C. Cultures were then split into two 5 ml cultures and grown at either 30°C or 16°C. Cells were then fixed by the addition of 700 μl of 37% formaldehyde and incubated at 30°C or 16°C for 90 min, and fluorescence in situ hybridization (FISH) using an oligo d(T) probe to detect poly(A) RNA was performed as described by Wong et al. (1997). Cells were also stained with DAPI to visualize DNA within the nucleus.

## Figures and Tables

**Table 1:** Statistical Data Relating to the Final Ensemble of Structures of NAB2 F5-F7

Structural Restraints	

NOE-derived distance restraints	
Intraresidue	2
Sequential	371
Medium (2 ≤ |i−j| ≤ 4)	132
Long (|i−j| > 4)	277
Ambiguous	76
Total	858
RDC restraints	
NH − N	61

Statistics for Accepted Structures	

Number of accepted structures	20
Mean AMBER energy terms (kcal mol^−1^ ± SD)	
E(total)	−3,805.9 ± 10.5
E(van der Waals)	−541.3 ± 9.1
E(distance restraints)	25.8 ± 3.0
Distance violations >0.2 Å (average per structure)	7.4 ± 1.7
Maximum distance violation	0.52 Å
Mean absolute RDC violation	1.85 ± 1.64 Hz
Rmsd from the ideal geometry used within AMBER	
Bond lengths	0.011 Å
Bond angles	2.46°

Ramachandran Statistics for Residues 410–480	

Most favored	89.0%
Additionally allowed	11.0%
Generously allowed	0.0%
Disallowed	0.0%

Average Atomic Rmsd to Mean Structure (±SD) for Residues 410–480	

N, C^α^, C′ atoms	0.69 ± 0.15 Å
All heavy atoms	1.18 ± 0.14 Å

**Table 2:** Characterization of *S. cerevisiae* Nab2 ZnF Variants

Nab2 Variant	ZnF	Growth			Affinity for A_8_ (μM)	Poly(A) Tail Length
		*ΔNAB2*	*dbp5 (rat8-2)*	*GFP-yra1-8*		
Wild-type		WT	−	−	0.5	WT
K416A	5	WT	−	+	15	WT
F417E	5	WT	+	++	18	+
C437S	6	cs	+++	+++	19	++
R438A	6	WT	++	−	58	+
E439F	6	WT	−	−	0.4	WT
F450A	6	WT	++	++	31	++
R459A	7	WT	−	−	7	WT
F460E	7	WT	−	+	28	WT
F417E+F460E	5+7	WT	++	±	71	++

The growth scale is − (no growth), ± (some modest growth in most concentrated spot), + (growth in most concentrated spot), ++ (growth in most concentrated and 1:10 dilution), and +++ (growth in most concentrated, 1:10 dilution, and 1:100 dilution). See also Table S1.

## Accession Numbers

Atomic coordinates and constraints for the NMR ensemble have been deposited in the PDB with accession code 2LHN, and the corresponding chemical shift assignments have been deposited in the BMRB with accession code 17858.

## Supplemental Information

Document S1. Figures S1–S3, Table S1, and Supplemental Experimental Procedures

Movie S1. The Structure of the Three Zn Fingers, Related to Figure 1
