# Supplementary material for: Structural Basis for Polyadenosine-RNA Binding by Nab2 Zn Fingers and Its Function in mRNA Nuclear Export
Source: Structure. 2012 Jun 6;20-540(6-7):1007–18. doi: 10.1016/j.str.2012.03.011 (PMC3384006; doi:10.1016/j.str.2012.03.011)
Supplement: Document S1. Figures S1–S3, Table S1, and Supplemental Experimental Procedures [file mmc1.pdf]

Structure, Volume *20*

Supplemental Information

Structural Basis for Polyadenosine-RNA Binding by Nab2 Zn Fingers and Its Function in mRNA Nuclear Export

Christoph Brockmann, Sharon Soucek, Sonja I. Kuhlmann, Katherine Mills-Lujan, Seth M. Kelly, Ji-Chun Yang, Nahid Iglesias, Francoise Stutz, Anita H. Corbett, David Neuhaus, and Murray Stewart

Inventory of Supplemental Information

Supplementary Methods: Detailed description of the analysis of the NMR data.

Supplementary Figure S1. Heteronuclear NOE for the ZnF5-7 fragment, plotted as a function of sequence, to illustrate the rigidity of the the-finger construct, related to Figure 1.

Supplementary Figure S2: ^{15}N-HMQC data for each finger, showing that the pattern of cross-peaks indicates that zinc-coordination by histidine is through $N^{\varepsilon 2}$, related to Figure 2.

Supplementary Figure S3: Control showing that poly(A) RNA localization is not altered in cells expressing any of the Nab2 zinc finger variants, related to Figure 7.

Supplementary Table S1: S. cerevisiae strains and plasmids used in this study, related to Table 2.

Supplementary references: For Supplementary Methods and for Table S1, showing sources of strains.

Supplementary Movie 1: Movie illustrating the structure of the three Zn fingers, related to Figure 1.

Supplemental Data

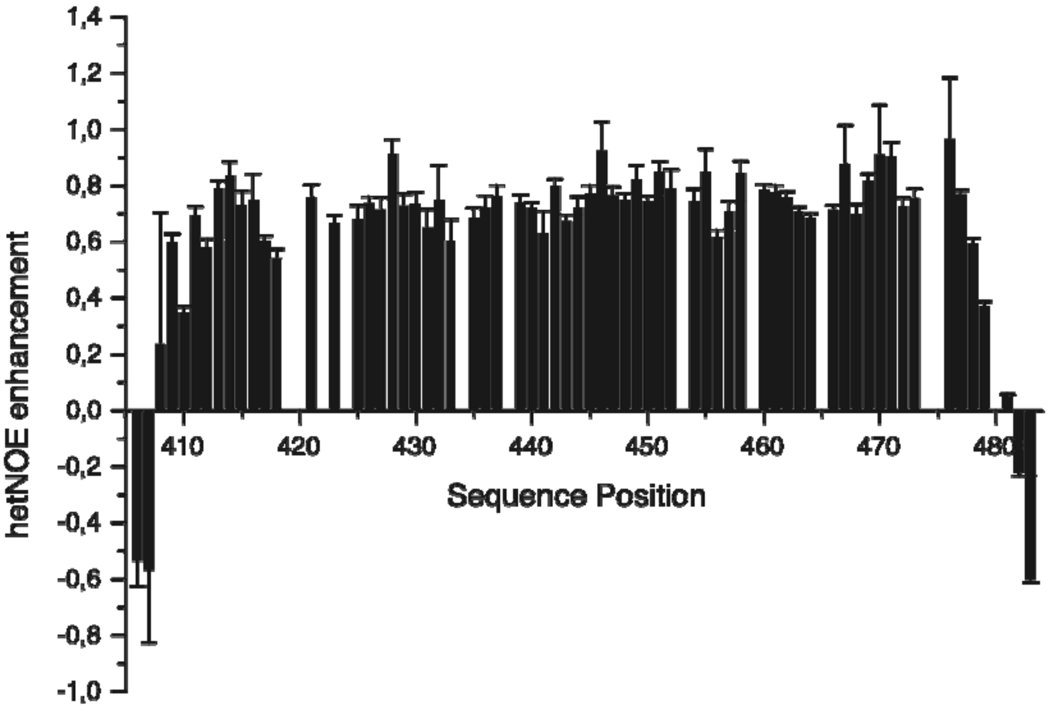

Supplemental Figure S1, related to Figure 1. Heteronuclear NOE for the ZnF5-7 fragment, plotted as a function of sequence. Apart from a small number of residues at each end of the construct (the N-terminal GPLGS cloning artefact and residues 481-483), the backbone rigidity as evidenced by the heteronuclear NOE data was essentially uniform along the entire ZnF5-7 fragment, with no additional flexibility evident in the inter-finger linker regions.

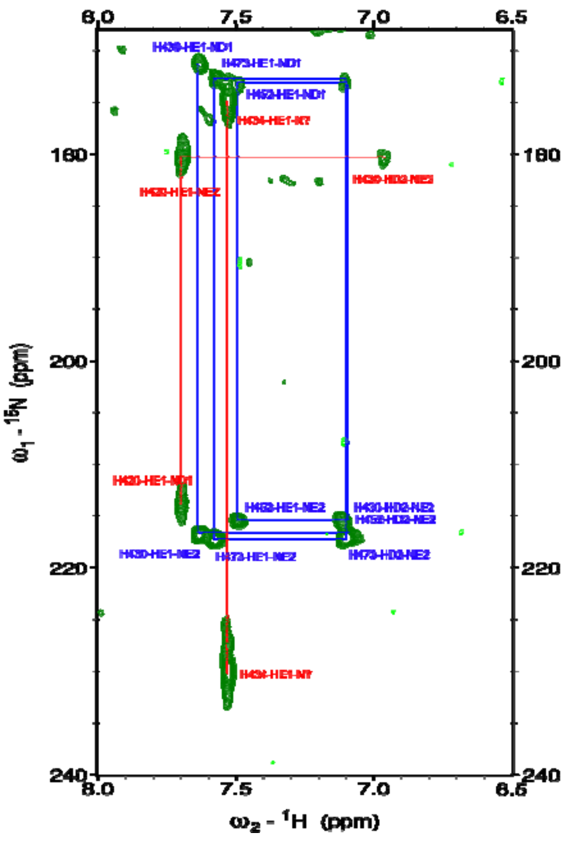

Supplementary Figure S2, related to Figure 2. ^{15}N-HMQC data. For each finger, the pattern of cross-peaks indicates that zinc-coordination by histidine is through $N^{\varepsilon 2}$.

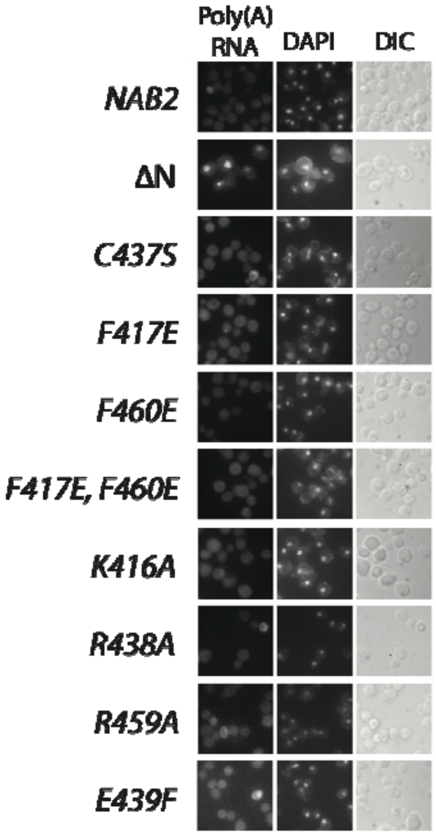

Supplementary Figure S3, related to Figure 7: Poly(A) RNA localization is not altered in cells expressing any of the Nab2 zinc finger variants. Bulk poly(A) RNA localization was analyzed by fluorescence in situ hybridization as described in the methods. In addition to visualization of poly(A) RNA, samples were also stained with DAPI to visualize the position of the nucleus and imaged by DIC imaging to show the position of the cells. Cells expressing the indicated variants of Nab2 as the sole copy of Nab2 were analyzed. Nuclear accumulation of poly(A) RNA was not observed in wild-type control (*NAB2*) or any zinc finger mutant analyzed (K416A, F417E, C437S, R438A, E439F, F450A, R459A, F460E, F417E+F460E) although, as control, nuclear accumulation of poly(A) RNA was clearly observed in the Nab2-ΔN variant.

Supplementary Movie Legend

Supplementary Movie 1, related to Figure 1: Structure of Nab2 Zn-fingers 5-7. Finger 5 is cyan, finger 6 is green, finger 7 is red, and peripheral residues at the N- and C-termini are grey. The side chains of the cysteine and histidine residues that chelate the Zn (gold sphere) in each finger are also shown. The three fingers form a single coherent structure in which they are arranged in an approximately helical configuration.

Supplementary Table

Table S1: S. cerevisiae strains and plasmids used in this study

Strain/Plasmid	Description	Source
ACY427	*ΔNAB2::HIS3* (pAC636) *MAT**a** leu2 ura3*	Green *et al.* 2002
ACY1669	*ΔNAB2::HIS3 rat8-2 (dbp5) MAT**a** leu2 ura3 trp1*	Tran *et al.* 2007
FSY2327 (ACY1497)	*ΔNAB2::HIS3 ΔYRA1::HIS3 MAT**α** leu2 ura3 trp1*	Iglesias *et al.* 2010
pRS315 (pAC3)	*CEN, LEU2*	Sikorskiand Hieter, 1989
pAC636	*NAB2, CEN, URA3*	Green *et al.* 2002
pAC717	*NAB2, CEN, LEU2*	Green *et al.* 2002
pFS2581 (pAC2266)	*GFP-yra1-8, CEN, TRP1*	Zenklusen *et al.* 2002 and this study
pFS2347	*GFP-YRA1 cDNA, CEN, TRP1*	Iglesias *et al.* 2010
pAC2307	*Nab2-C437S, CEN, LEU2*	Kelly *et al.* 2007
pAC2620	*Nab2-K416A, CEN, LEU2*	This study
pAC2877	*Nab2-F417E, CEN, LEU2*	This study
pAC2618	*Nab2-R438A, CEN, LEU2*	This study
pAC2703	*Nab2-E439F, CEN, LEU2*	This study
pAC2503	*Nab2-F450A, CEN, LEU2*	Kelly *et al.* 2010
pAC2647	*Nab2-R459A, CEN, LEU2*	This study
pAC2878	*Nab2-F460E, CEN, LEU2*	This study
pAC2924	*Nab2-F417E F460E, CEN, LEU2*	This study

Supplemental Materials and Methods:

NMR spectroscopy and structure calculations

An essentially complete set of resonance assignments was made using a standard suite of triple resonance NMR experiments, and structural constraints were derived from NOESY and RDC data. For experiments used to derive structural constraints, samples contained 1.2 mM ^{15}N, ^{13}C-labelled solutions of Nab2 409-483. The following spectra were acquired: 2D: [^{15}N-^{1}H] HSQC, long-range-optimised [^{15}N-^{1}H] HMQC to correlate His ring ^{1}H and ^{15}N signals,[2] [^{13}C-^{1}H] HSQC covering the full ^{13}C spectral width, constant-time [^{13}C-^{1}H] HSQC covering only the aliphatic ^{13}C region, constant-time [^{13}C-^{1}H] HSQC covering only the aromatic ^{13}C region; 3D data sets: CBCANH (Grzesiek 1992), CBCACONH (Grzesiek 1992), HBHACONH (Grzesiek 1993), [^{1}H-^{13}C-^{1}H] HCCH-TOCSY (Kay 1993), [^{13}C-^{13}C-^{1}H] HCCH-TOCSY (Kay 1993), ^{15}N NOESY-HSQC (τ_m = 120 ms and τ_m = 50 ms) (Sklenar 1993), ^{13}C NOESY-HSQC (τ_m = 150 ms; separate datasets acquired for ^{13}C aliphatic and aromatic spectral regions) (Davis 1992). Residual dipolar couplings were measured using a 0.6 mM ^{15}N, ^{13}C-labelled solution of Nab2 409-483, to which Tobacco Mosaic Virus was added to a final concentration of 25 mg/ml; splittings were measured in F_1 cross-sections of [^{15}N-^{1}H] HSQC IPAP spectra (Ottiger 1998). Spectra were processed using the program TOPSPIN (Bruker GmbH, Karlsruhe) and analysed using the program CCPN analysis (Vranken 2005).

Initial structures for the free proteins were calculated using the semi-automatic program CYANA (Herrmann 2002), for which the input comprised the protein sequence, the full resonance assignment and the following 3D NOESY datasets: ^{15}N NOESY-HSQC (τ_m = 120 ms), ^{13}C aliphatic region NOESY-HSQC (τ_m = 150 ms) and ^{13}C aromatic region NOESY-HSQC (τ_m = 150 ms). During the CYANA calculations no metal was represented

explicitly, but the effect of metal binding was approximated by including inter-ligand distance constraints as follows: S^{γ} to S^{γ}, 3.7-4.0Å; S^{γ} to His-N, 3.4-3.8Å; His-N to His-N, 3.1-3.5Å. The pattern of zinc connectivities to the His residues was established using long-range ^{15}N-HMQC experiments as described by Legge *et al.* (2004), which showed unambiguously that the $N^{\varepsilon 2}$ atom binds the zinc in all three cases (see Supplementary Figure S2).

To refine these structures, and to allow explicit Zn bonding and geometry terms to be employed in the force-field, we next calculated structures using XPLOR-NIH (Schwieters *et al.* 2003). The set of NOE restraints generated by CYANA were re-imported into CCPNMR Analysis and curated manually during many iterative cycles of structure calculation, carefully analyzing violations and assigning ambiguous NOE cross-peaks on the basis of distances in the current structural round (avoiding bias towards known structures). Since the XPLOR-NIH calculations employed r^{-6} summation for equivalent and non-stereospecifically assigned groups, and since no stereoassignments were made (and the stereoassignment-swapping protocol within XPLOR-NIH was not applied), the constraints for all such groups were converted to group constraints (i.e. such groups were specified using wildcards such as HB*). All lower bounds were set to zero (Hommel *et al.* 1992). Structures were calculated from polypeptide chains with randomized ϕ and ψ torsion angles using a two-stage simulated annealing protocol within XPLOR-NIH, essentially as described elsewhere (Muto *et al.* 2004), but employing larger numbers of cycles as follows: first-stage calculations comprised Powell energy minimization (500 steps), dynamics at 1000K (25000 steps), increase of the van der Waals force constant and tilting of the NOE potential function asymptote (4000 steps), switching to a square-well NOE function then cooling to 300K in 2000 step cycles, and final Powell minimization (1000 steps). Second-stage calculations used Powell

minimization (500 steps), annealing with a strong van der Waals force constant and square-well NOE potential function, cooling to 300K in 1000 step cycles, followed by 2000 steps of Powell minimisation.

The structures calculated in XPLOR-NIH were finally subjected to a further stage of refinement using a full force field and an implicit water-solvent model as implemented in the program AMBER 9 (Case 2009). Calculations comprised initial minimization (200 steps steepest descent then 1800 steps conjugate gradient), then two rounds of 20ps of simulated annealing (each comprising 5000x1fs-steps heating from 0 - 500K; 13000x1fs-steps cooling to 100K; 2000x1fs-steps cooling to 0K) and final minimization (200 steps steepest descent then 1800 steps conjugate gradient). The experimental distance and amide NH RDC-derived restraints were applied throughout, and force constants for the distance restraints were increased linearly during the simulated annealing to a final value of 20 kcal mol^{-1} $Å^{-2}$. The weighting of the RDC terms (dwt) was set to 0.02 until the final minimization, when it was increased to 0.05; Saupe order matrix terms from the final step of each stage were transferred to the input of the succeeding stage using scripts. Implicit solvent representation using the generalized Born method (Xia 2002) was employed throughout (igb=1), and Langvin temperature control was used (ntt=3; gamma_ln=5). The program CLUSTERPOSE was used to calculate the mean rmsd of ensembles to their mean structure (Diamond 1995) and structures were visualized using the program PYMOL (Schroedinger, LLC).

Supplemental references

Davis, A.L., Keeler, J., Laue, E.D., and Moskau, D.J. (1992). Experiments for recording pure-absorption heteronuclear correlation spectra using pulsed field gradients. J. Magn. Reson. *98*, 207-216.

Green, D.M. *et al.* (2002). Nab2p is required for poly(A) RNA export in *Saccharomyces cerevisiae* and is regulated by arginine methylation via Hmt1p. J. Biol. Chem. *277*, 7752-7760.

Grzesiek, S. and Bax, A. (1992). Correlating backbone amide and side chain resonances in larger proteins by multiple relayed triple resonance NMR. J. Am. Chem. Soc. *114*, 6291-6293.

Grzesiek, S. and Bax, A. (1993). Amino-acid type determination in the sequential assignment procedure of uniformly $^{13}C/^{15}N$-enriched proteins. J. Biomol. NMR, *3*, 185-204

Hommel U, Harvey TS, Driscoll PC & Campbell ID (1992) Human epidermal growth factor. High resolution solution structure and comparison with human transforming growth factor alpha. J. Mol. Biol. *227*, 271-282.

Iglesias, N., Tutucci, E., Gwizdek, C., Vinciguerra, P., Von Dach, E., Corbett, A.H., Dargemont, C.and Stutz, F. (2010). Ubiquitin-mediated mRNP dynamics and surveillance prior to budding yeast mRNA export. Genes Dev. *24*, 1927-1938.

Kelly, S.M. *et al.* (2007). Recognition of polyadenosine RNA by zinc finger proteins. Proc. Natl. Acad. Sci. USA *104*, 12306-12311.

Kelly, S.M. *et al.* (2010). Recognition of polyadenosine RNA by the zinc finger domain of nuclear poly(A) RNA-binding protein 2 (Nab2) is required for correct mRNA 3'-end formation. J. Biol. Chem. *285*, 26022-26032.

Legge GB, Martinez-Yamout MA Hambly DM, Trinh T, Lee BM, Dyson HJ, *et al.* (2004). ZZ domain of CBP: an unusual zinc finger fold in a protein interaction module. J. Mol. Biol. *343*, 1081-1093.

Muto Y Pomeranz-Krummel D, Oubridge C, Hernandez H, Robinson CV, Neuhaus D & Nagai K (2004) The structure and biochemical properties of the human spliceosomal protein U1C. J. Mol. Biol. *341*, 85-98.

Ottiger, M., Delaglio, F. and Bax, A.D. (1998). Measurement of J and dipolar couplings from simplified two-dimensional NMR spectra. J. Magn. Reson. *131*, 373-378.

Sikorski, R.S. and Hieter, P. (1989). A system of shuttle vectors and yeast host strains designed for efficient manipulation of DNA in *Saccharomyces cerevisiae*. Genetics, *122*, 19-27.

Sklenar, V., Piotto, M., Leppik, R. and Saudek, V. (1993). Gradient-tailored water suppression for ^{1}H-^{15}N HSQC experiments optimized to retain full sensitivity. J. Magn. Reson. *A 102*, 241-245.

Tran, E.J., Zhou, Y., Corbett, A.H., and Wente, S.R. (2007). The DEAD-box protein Dbp5 controls mRNA export by triggering specific RNA:protein remodeling events. Mol. Cell *28*, 850-859.

Vinciguerra, P., Iglesias, N., Camblong, J., Zenklusen, D., and Stutz, F. (2005). Perinuclear Mlp proteins downregulate gene expression in response to a defect in mRNA export. EMBO J. *24*, 813-823.

Vranken, W.F., Boucher, W., Stevens, T.J., Fogh, R.H., Pajon, A., Llinas, M., Ulrich, E.L., Markley, J.L., Ionides, J. and Laue, E.D. (2005). The CCPN Data Model for NMR Spectroscopy: Development of a Software Pipeline. Proteins *59*, 687-696.

Zenklusen, D., Vinciguerra, P., Wyss, J.C., and Stutz, F. (2002). Stable mRNP formation and export require cotranscriptional recruitment of the mRNA export factors Yra1p and Sub2p by Hpr1p. Mol. Cell, Biol. *22*, 8241-8253.
