# Supplementary figures and images for: Structural Basis for Polyadenosine-RNA Binding by Nab2 Zn Fingers and Its Function in mRNA Nuclear Export
Source: Structure. 2012 Jun 6;20-540(6-7):1007–18. doi: 10.1016/j.str.2012.03.011 (PMC3384006; doi:10.1016/j.str.2012.03.011)

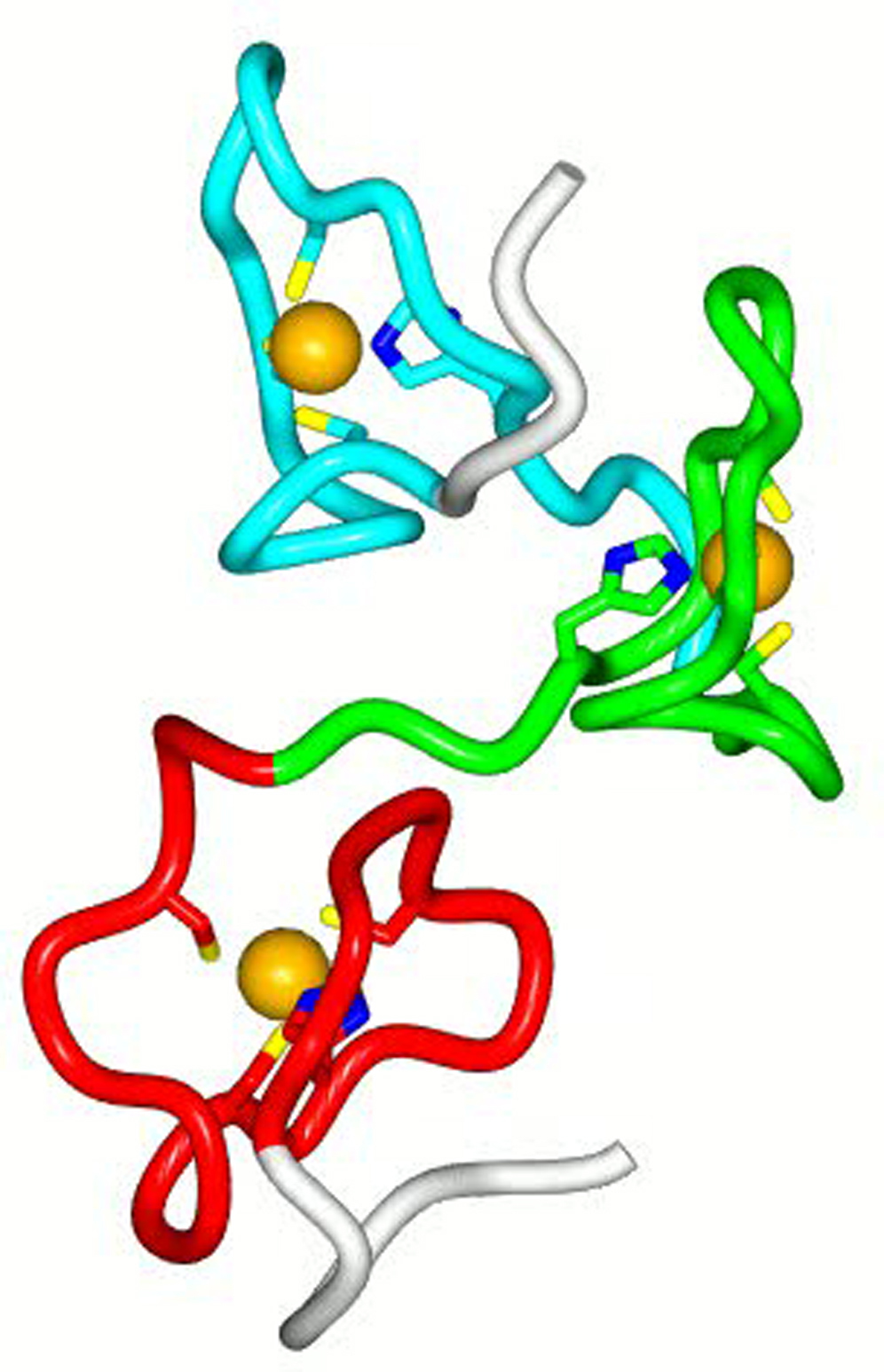

Supplement: Movie S1. The Structure of the Three Zn Fingers, Related to Figure 1 [file mmc2.jpg]
